# Long-Term Immune Recovery After Hematopoietic Stem Cell Transplantation for ADA Deficiency: a Single-Center Experience

**DOI:** 10.1007/s10875-021-01145-w

**Published:** 2021-10-16

**Authors:** Alexandra Y. Kreins, Helena F. Velasco, Kai-Ning Cheong, Kanchan Rao, Paul Veys, Austen Worth, H. Bobby Gaspar, Claire Booth

**Affiliations:** 1 Department of Immunology and Gene Therapy, Great Ormond Street Hospital for Children NHS Foundation Trust, London, UK; 2 UCL Great Ormond Street Institute of Child Health, London, UK; 3 Department of Pediatric Allergy and Immunology, Federal University of São Paolo, São Paolo, Brazil; 4 Department of Paediatric Rheumatology and Immunology, Hong Kong Children’s Hospital, Hong Kong, Hong Kong; 5 Department of Bone Marrow Transplantation, Great Ormond Street Hospital for Children NHS Foundation Trust, London, UK; 6 Orchard Therapeutics, London, UK

**Keywords:** ADA SCID, Hematopoietic stem cell transplantation, Reduced intensity conditioning, Primary immunodeficiency

## Abstract

Unconditioned hematopoietic stem cell transplantation (HSCT) is the recommended treatment for patients with adenosine deaminase (ADA)-deficient severe combined immunodeficiency with an HLA-matched sibling donor (MSD) or family donor (MFD). Improved overall survival (OS) has been reported compared to the use of unrelated donors, and previous studies have demonstrated that adequate cellular and humoral immune recovery can be achieved even in the absence of conditioning. Detailed insight of the long-term outcome is still limited. We aim to address this by studying a large single-center cohort of 28 adenosine deaminase-deficient patients who underwent a total of 31 HSCT procedures, of which more than half were unconditioned. We report an OS of 85.7% and event-free survival of 71% for the entire cohort, with no statistically significant differences after procedures using related or unrelated HLA-matched donors. We find that donor engraftment in the myeloid compartment is significantly diminished in unconditioned procedures, which typically use a MSD or MFD. This is associated with poor metabolic correction and more frequent failure to discontinue immunoglobulin replacement therapy. Approximately one in four patients receiving an unconditioned procedure required a second procedure, whereas the use of reduced intensity conditioning (RIC) prior to allogeneic transplantation improves the long-term outcome by achieving better myeloid engraftment, humoral immune recovery, and metabolic correction. Further longitudinal studies are needed to optimize future management and guidelines, but our findings support a potential role for the routine use of RIC in most ADA-deficient patients receiving an HLA-identical hematopoietic stem cell transplant, even when a MSD or MFD is available.

**Supplementary Information:**

The online version contains supplementary material available at 10.1007/s10875-021-01145-w.

## Introduction

Adenosine deaminase (ADA) deficiency results in one of the more common forms of autosomal recessive severe combined immunodeficiency (SCID) [1, 2]. ADA is an enzyme in the purine metabolism. More than 70 disease-causing mutations have been described, leading to an impaired purine salvage pathway with accumulation of toxic metabolites (adenosine, 2’deoxyadenosine, and deoxyadenosinetriphosphate (dATP)), as well as inactivation of S-adenosylhomocysteine hydrolase (SAHase) [3]. ADA expression is particularly high in lymphoid tissues, and therefore, ADA deficiency leads to severe abnormalities in lymphoid development [4–6]. It is typically associated with lymphopenia with abnormal T, B, and natural killer (NK) cell numbers and function. Patients usually present with failure to thrive and severe or recurrent opportunistic infections. Additionally, patients display typical non-immunological features, including sensorineural hearing loss [7, 8], non-infectious pneumonitis, and pulmonary alveolar proteinosis [9, 10], skeletal dysplasias [11–14], urogenital abnormalities [15], hepatic dysfunction [16], cognitive impairment, and behavioral abnormalities [17, 18]. This variety of clinical manifestations can be explained by the metabolic pathogenesis affecting multiple organs and tissues [3]. The immune system is the most severely affected, and without treatment, ADA-deficient individuals die in their first or second year of life from infective causes. Early intervention is thus critical. Unlike other forms of SCID, a number of options are available for the treatment of ADA-SCID [1, 19, 20], namely hematopoietic stem cell transplantation (HSCT) [21], enzyme replacement therapy (ERT) with PEG-ADA [22], and more recently gene therapy (GT) where both licensed and experimental therapies are now available [23–26]. Importantly, the survival associated with GT, which is currently 100%, invites comparison with HSCT outcomes.

Several single-center [27–30] and multicenter studies [21, 31, 32] have highlighted the efficiency of HSCT, in particular from HLA-matched sibling donors (MSDs) and HLA-matched family donors (MFDs), providing long-term correction of the immune and metabolic abnormalities in ADA-deficient patients. The largest study to date is a multicenter study investigating the outcome of HSCT in 106 ADA-deficient SCID patients [21]. Fifty-four patients included in this study received a MSD or MFD HSCT between 1981 and 2009. These patients showed a better overall survival (OS) (86% and 81%, respectively) than patients who underwent a transplant procedure from HLA-matched unrelated donors (MUDs), haploidentical donors, or HLA-mismatched unrelated donors (MMUD) (67%, 43%, and 29%, respectively). Most HSCT procedures with MSD/MFD were unconditioned, i.e., without reduced intensity conditioning (RIC) or myeloablative conditioning (MAC). However, these data and data from other ADA specific studies were collected from patients as far back as 1981. As such, these data are not reflective of current HSCT practices and outcomes and may not be an appropriate comparison for the more recently conducted GT studies. Additionally, data on immune reconstitution and other parameters of immune recovery, such as discontinuation of immunoglobulin replacement treatment (IgRT), were not available for many patients included in these studies. With this in mind, we sought to collect data from a more contemporaneous ADA patient cohort, specifically from a center undertaking both HSCT and GT with similar standards of patient care and monitoring. This report therefore documents the outcome of all patients with ADA SCID who have undergone HSCT from 2000 onwards at a single center. To our knowledge, this is the largest single-center cohort to date.

## Methods

We undertook a retrospective analysis of the outcome of all HSCT procedures performed for ADA-deficient SCID at Great Ormond Street Hospital (GOSH), London, since 2000. Thirty-one HSCT procedures were performed in 28 patients between September 2001 and January 2019. Transplant procedures in 12 patients happened before March 2009, and analysis of their outcome was included in a previous multicenter study [21]. We report more detailed outcomes for these patients together with the first description of 16 additional patients, who have been transplanted after March 2009. ADA deficiency was diagnosed by impaired or absent ADA activity and was confirmed by genetic testing. Before HSCT, all patients received supportive care, including antimicrobial prophylaxis, IgRT, and in most cases ERT with PEG-ADA. Upon referral, all patients were assessed for HSCT at GOSH and were managed there immediately after HSCT. Long-term follow-up was shared with local centers in the UK and abroad. Each patient included in this study had at least 2 years follow-up at GOSH after transplantation, except two, of whom one was last assessed from a laboratory point of view at GOSH at 0.9 years post-transplantation and one was transplanted in January 2019. This second patient had 1 year follow-up up to the endpoint for data collection. Data was retrieved from center-based electronic patient records and was stored anonymously. Pre-transplant data, such as gender, ADA activity pre-ERT when available, initial clinical presentation, and comorbidities at the time of transplantation, were collected for all patients. Characteristics of the transplant procedures were gathered including patient age at the time of transplantation, donor source, tissue-typing results, stem cell source, and conditioning details. Matched donors were defined as displaying a 10/10 match in tissue type with the recipient. We assessed survival time after HSCT up to January 2020 and, where applicable, cause of death. Kaplan–Meier curves were used to analyze overall and event-free survival (OS and EFS) in SPSS (events are defined as death, administration of an additional cell infusion from the same donor or the need for a second HSCT procedure using a different donor). Three patients received a second transplant, and one patient underwent HSCT after previously having received HSC-GT. For these four patients, survival was calculated from the last treatment received. For the two patients who received a second infusion from the same donor, survival was calculated from the date of the initial procedure. The log rank test was used to compare the survival distributions between subgroups differentiating between either donor source or type of conditioning. Transplant-related morbidity, in particular the incidence of acute and chronic graft-versus-host disease (GVHD), was analyzed. Laboratory results to evaluate immune reconstitution post-HSCT were collected at 6 months (± 1 month) and 12 months (± 1 month) after transplantation, as well as at the time of the last follow-up. Given the small cohort size, comparison of outcomes between subgroups was analyzed using descriptive statistics, with results plotted in Python using box plots. We also collected information on donor engraftment and, when available, ADA activity at the time of the last evaluation, as well as information regarding interruption of IgRT and vaccine responses at 2 years post-HSCT. For continuous variables, Kruskal–Wallis and Mann–Whitney tests were used to compare outcomes between subgroups. For categorical variables, chi-square and Fisher’s exact tests were used to assess differences in outcomes between subgroups.

## Results

A total of 28 ADA-deficient SCID patients (17 males and 11 females) underwent 31 transplant procedures at GOSH between 2000 and 2019. Patient characteristics pre-HSCT are summarized in Table 1. Twelve patients suffered from infections before transplant, of which half were receiving treatment for active infections at the time of transplantation. The median age at the time of transplantation was 12.5 months. The youngest patient was under a month old when transplanted, and the oldest patient, who had previously been treated by GT, was 119.6 months old. At least 7 patients (25%) did not receive ERT pre-HSCT. There was no significant difference in the age at transplant of those receiving ERT. Post-HSCT data was collected over a cumulative total of 209.9 years of follow-up after the 31 transplant procedures. The median follow-up after HSCT was 6.3 years with the longest follow-up period covering 17.4 years. The follow-up period for each procedure was determined from the time of transplantation to the time of the most recent laboratory assessment at GOSH up to 01/2020, the time of a second HSCT procedure, or the time of death. For this study, five patients, who continue to be monitored abroad by their referring centers, have been considered lost to follow-up after the last assessment of their immune reconstitution at GOSH.

**Table 1:** Patient details before transplantation

Patient	Sex	Nr of procedures	Age at HSCT (mo)	ADA activity (nmol/mg Hb/h)	dATP level at presentation (mmol/L)	Initial presentation	Comorbidities at time of transplantation
1	F		2.2	0	Increased	Respiratory distress	Disseminated CMV disease
2	F		1.6	14	319	Positive family history	No active problems
3	F		6.5	< 1	Increased	Positive family history, recurrent infections, FTT	Adenovirus and Rotavirus gastroenteritis, Parainfluenza 3 LRTI, anti-BCG prophylaxis
4	M		3.6	0	1305	NA	NA
5	M		2.5	2	Increased	Skin rash, cytopenias	Anti-BCG prophylaxis
6	F		13	NA	NA	Positive family history	CMV reactivation, Adenovirus viremia
7	M	2 infusions	1.4 and 11.9	3	NA	Positive family history, FTT	Pneumonia and sepsis
8	M		3.4	0	1034	Recurrent respiratory illnesses	ADA lung, anti-BCG prophylaxis
9	F		2.5	0	Increased	FTT, recurrent thrush	Rotavirus in stool, E. Coli UTI, ADA lung
10	M		17.6	NA	NA	Recurrent respiratory infections	Bronchiectasis, anti-BCG prophylaxis
11	F	2 infusions	16.1 and 30.1	0	1158	Respiratory illnesses	ADA lung
12	F		12.5	18	590	Respiratory failure, thrombocytopenia, infantile hemangiomatosis	Anti-BCG prophylaxis, Oseltamivir prophylaxis after recent influenza B infection, Rotavirus in stool, ADA lung
13	F		14.5	NA	NA	FTT	No active problems
14	M		12.1	Impaired	Increased	Respiratory distress, oral thrush rash	No active problems
15	M		17.3	0	12	Positive family history, FTT, oral thrush, facial rash	No active problems
		2^nd^ HSCT	30	Impaired	280		No active problems
16	F		5.4	0	358	FTT, skin rash	No active problems
17	F		21.7	0	670	Positive family history	No active problems
18	M		16.8	0	546	Positive family history	Anti-BCG prophylaxis, ADA lung
19	M		4.1	21	344	Respiratory illnesses, FTT	ADA lung
20	M	Previous GT	119.6	NA	NA	Recurrent infections, FTT	Hypocellular and dysplastic marrow with cytopenias secondary to previous GT
21	M		12.9	0	1671	Respiratory distress, FTT	No active problems
22	M		22.9	6	427	Positive family history	No active problems
23	M		16.7	0	246	Recurrent respiratory infections	Anti-BCG prophylaxis, bronchiectasis, Adenovirus/HSV/HHV6 viremias
24	M		37.9	1	119	Recurrent infections	No active problems
		2^nd^ HSCT	62.9	3	287		Hypocellular marrow with cytopenias secondary to ERT
25	M		4.6	3	1663	Recurrent respiratory infections, FTT	LRTI (H. influenza), thrombocytosis secondary to ERT
		2^nd^ HSCT	20.7	41	< 50		Skin and liver GVHD, hypertrophic cardiomyopathy, chronic lung disease, HHV6 viremia, NPA + with Rhinovirus
26	M		0.9	0	1235	Positive family history	Pneumonia
27	F		7.4	23	< 50	Disseminated CMV disease	CMV prophylaxis with valganciclovir
28	M		6.8	0	1299	FTT, recurrent infections	Rotavirus gastroenteritis, anti-BCG prophylaxis, thrombocytosis secondary to ERT

*NA* data not available, *FTT* failure to thrive, *LRTI* lower respiratory tract infection, *UTI* urinary tract infection

### Survival Outcomes

**Fig. 1:**
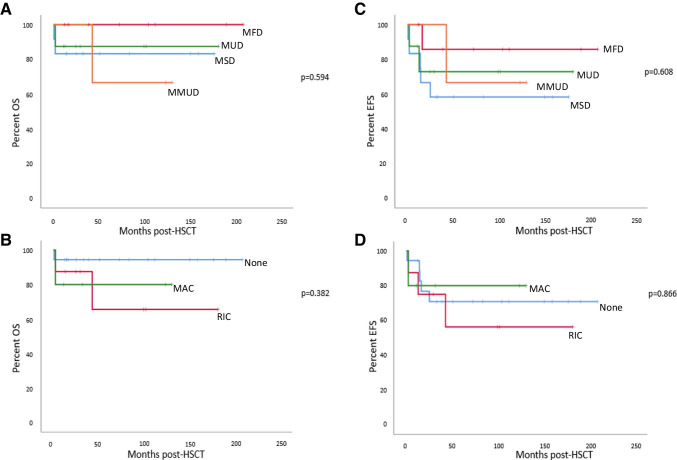
Overall survival and event-free survival after HSCT: Censored Kaplan–Meier curves showing OS in relation to donor source (**A**) and intensity of conditioning (**B**) and EFS in relation to donor source (**C**) and intensity of conditioning (**D**). For OS, *n* = 28 subjects, overall OS = 85.7%; whereas for EFS, n = 31 HSCT procedures, with 3 of the 28 subjects having received 2 HSCT procedures each, overall EFS = 71%

The OS for the 28 patients at the time of analysis was 85.7% (*n* = 24) (Fig. 1A–B), accounting for the death of 4 patients post-HSCT (Supplementary Table 1). One patient died of pre-existing disseminated CMV disease 16 days post-HSCT. A second death occurred 56 days post-HSCT due to sepsis. A third patient, who had previously received unsuccessful gammaretroviral GT, died 3.6 years after HSCT in the context of lung GVHD exacerbation. The fourth death was in a patient who developed chronic GVHD (cGVHD) after a first transplant procedure. A second HSCT was performed in an attempt to treat his cGVHD, but he died 55 days later due to progressing respiratory failure.

The EFS, where events were defined as death or need for a second procedure, was 71% (*n* = 22) for all 31 HSCT procedures in the 28 patients (i.e., for 3 individuals who underwent 2 HSCT procedures, both procedures were included) (Fig. 1C–D). Two patients who received unconditioned MSD HSCT required a top up with infusion of whole bone marrow, at 14.5 and 14 months after the first transplant. Three patients required a second, conditioned, transplant because of absent or poor engraftment, which occurred respectively 12.7, 25.0, and 16.1 months after the first HSCT procedures (2 of which were unconditioned and one performed after RIC). In the months preceding the second HSCT procedure, 2 patients had resumed ERT.

### Outcomes Related to Donor Source and Type of Conditioning

Details of all the HSCT procedures are specified in Table 2. In summary, MSDs were the most frequent donor source (*n* = 12, 39%), followed by MFDs (*n* = 8, 26%) and MUDs (*n* = 8, 26%). Three procedures used MMUDs (9%). No haploidentical transplant procedures were performed. Eighteen (58%) transplant procedures were stem cell infusions without prior conditioning, although one patient received serotherapy with alemtuzumab in the context of residual T cell immunity. These unconditioned infusions included all procedures using MSDs and MFDs, except 2, which were second transplantations using MAC. Three other procedures employed MAC, and the remaining 8 procedures were performed following RIC; all using unrelated donors. Outcome post-HSCT was analyzed in relation to the type of transplant procedure (Fig. 1). OS was higher in matched transplant settings (100% after MFD, 87.5% after MUD and 83.3% after MSD HSCT), compared to survival after mismatched transplant (66.7% after MMUD HSCT). These differences were not statistically significant (*p* = 0.594). Unconditioned procedures had a higher OS (93.8%) than those after RIC (71.4%); however, this was not statistically significant either (*p* = 0.382). Pairwise comparisons between the different donor sources or between the different types of conditioning again were not statistically different. Acute GVHD (aGVHD) occurred after half of the procedures (Table 3). This affected 11.1% of the patients after MSD HSCT procedure, whereas it was observed after 75%, 50%, and 100% of procedures with MFD, MUD, and MMUD, respectively. The incidence of aGVHD was similar in patients who received an unconditioned procedure and those receiving RIC (47.1% and 42.9%, respectively, *p* = 0.50). Four patients (18.2%) suffered from cGVHD, including 2 patients who died (Table 3). Three of these patients had received an unconditioned HSCT from a MFD. The fourth had a MMUD procedure after MAC.

**Table 2:** HSCT procedure details

HSCT Nr *(patient nr)*	Donor source	HLA matching	Conditioning	Stem cell source	Outcome	Years of follow-up
1 *(1)*	MSD	10/10	None	BM	Died	0.0
2 *(2)*	MFD	10/10	None	BM	Alive	17.4
3 *(3)*	MUD	12/12	RIC (fludarabine/melphalan/alemtuzumab)	BM	Died	0.2
4 *(4)*	MFD	10/10	None	BM	Alive	15.9
5 *(5)*	MSD	10/10	None	BM	Alive	14.8
6 *(6)*	MUD	10/10	RIC (fludarabine/melphalan/alemtuzumab)	BM	Alive	15.1
7 *(7)*	MSD	10/10	None	BM	Alive	14.1
8 *(8)*	MSD	10/10	None	BM	Alive	13.3
9 *(9)*	MFD	10/10	None	BM	Alive	13.1
10 *(10)*	MSD	10/10	None	BM	Alive	^&^5.5
11 *(11)*	MSD	10/10	None	CB	Alive	11.3
12 *(12)*	MMUD	8/10 (2Cmm)	MAC (treosulfan/cyclophosphamide)	CB	Alive	10.4
13 *(13)*	MMUD	9/10 (1DQmm)	MAC (treosulfan/cyclophosphamide)	CB	Alive	9.6
14 *(14)*	MUD	10/10	MAC (treosulfan/cyclophosphamide)	CB	Alive	^&^0.9
15 *(15)*	MUD	10/10	RIC (fludarabine/treosulfan)	CB	Received 2^nd^ HSCT	1.1
16 *(16)*	MFD	10/10 (11/12)	None	BM	Alive	9.3
17 *(17)*	MUD	10/10	RIC (fludarabine/melphalan/alemtuzumab)	PBSC	Alive	^&^2.4
18 *(18)*	MFD	10/10 (11/12)	(Alemtuzumab)	BM	Alive	^&^3.2
19 *(19)*	MFD	10/10	None	BM	Alive	8.7
20 *(20)*	MMUD	9/10 (1Cmm)	RIC (fludarabine/melphalan/alemtuzumab)	PBSC	Died	3.6
21 *(21)*	MUD	10/10	RIC (fludarabine/melphalan/alemtuzumab)	PBSC	Alive	8.5
*22 *(15)*	MUD	10/10	RIC (fludarabine/melphalan/alemtuzumab)	PBSC	Alive	8.3
23 *(22)*	MUD	10/10	RIC (fludarabine/melphalan/alemtuzumab)	PBSC	Alive	^&^2.0
24 *(23)*	MSD	10/10	None	BM	Alive	7.0
25 *(24)*	MSD	10/10	None	BM	Received 2^nd^ HSCT	2.1
26 *(25)*	MFD	10/10 (11/12)	None	BM	Received 2^nd^ HSCT	1.3
27 *(26)*	MSD	10/10	None	BM	Alive	4.2
*^†^28 *(25)*	MSD	12/12	MAC (fludarabine/treosulfan/thiotepa/ATG)	BM	Died	0.2
29 *(27)*	MSD	10/10	None	BM	Alive	2.8
*30 *(24)*	MSD	10/10	MAC (fludarabine/treosulfan/thiotepa)	BM	Alive	2.6
31 *(28)*	MFD	12/12	None	PBSC	Alive	1.0

^*^Second HSCT procedure; †T-cell depleted HSCT procedure; ^&^lost to follow-up

**Table 3:** Monitoring post-HSCT

HSCT Nr (patient nr)	HSCT Details	aGVHD (grade)	cGVHD	TREC levels	Normal TCR Vβ repertoire	IgRT stopped	ADA activity (nmol/mg Hb/h)	dATP levels (μmol/L)*	Other complications
1 (1)	Uncond. MSD	-	-	-	-	-	-	-	-
2 (2)	Uncond. MFD	Gut (III)	No	Low	No	Yes	2	< 50	Hearing loss, learning issues
3 (3)	RIC MUD	-	-	-	-	-	-	-	-
4 (4)	Uncond. MFD	Skin, gut (III)	Scleroderma Vitiligo	NA	NA	Yes	31	< 50	Diabetes
5 (5)	Uncond. MSD	Skin, gut (II)	No	Low	NA	No	18	< 50	Hearing loss, learning issues
6 (6)	RIC MUD	No	No	Low	Yes	Yes	57	< 50	AIHA
7 (7)	Uncond. MSD	No	No	Normal	Yes	Yes	5	NA	ADHD, ketotic hypoglycemia, toe walking
8 (8)	Uncond. MSD	No	No	Very low	Yes	No	3	< 50	None
9 (9)	Uncond. MFD	Skin (II)	Scleroderma	Negligible	Yes	Yes	NA	NA	AI thyroiditis, hearing loss
10 (10)	Uncond. MSD	No	No	NA	NA	Yes	NA	NA	None
11 (11)	Uncond. MSD	No	No	Low	Yes	Yes	5	< 50	Delayed menarche
12 (12)	MAC MMUD	Skin (III)	No	Normal	Yes	Yes	72	< 50	Hypothyroidism, precocious puberty, hearing loss, acanthosis nigricans, developmental delay
13 (13)	MAC MMUD	Skin (?)	No	Normal	Yes	Yes	68	< 50	Hearing loss, developmental delay
14 (14)	MAC MUD	Skin (II)	NA	NA	NA	Yes	NA	< 50	NA
15 (15)	RIC MUD	No	No	-	-	-	-	-	-
16 (16)	Uncond. MFD	No	No	Low	Yes	Yes	37	NA	Hypothyroidism, hearing and learning issues
17 (17)	RIC MUD	No	NA	NA	No	Yes	NA	NA	NA
18 (18)	Uncond. MFD	Skin (I)	NA	NA	No	Yes	NA	NA	NA
19 (19)	Uncond. MFD	Skin (I)	No	Very low	Yes	No	1	157	Hearing loss, hypothyroidism
20 (20)	RIC MMUD	Skin (III)	Skin, lung, scleroderma	-	NA	No	NA	NA	Renal impairment (2ndary to CSA), myocardial dysfunction post GT
21 (21)	RIC MUD	No	No	Normal	Yes	Yes	43	< 50	ADHD, hearing loss
22 (15)	RIC MUD	Skin (I)	No	Low	Yes	Yes	130	NA	Hearing loss
23 (22)	RIC MUD	Skin (II)	No	NA	NA	Yes	NA	NA	NA
24 (23)	Uncond. MSD	No	No	Low	Yes	Yes	27	< 50	Bronchiectasis, chronic wheeze
25 (24)	Uncond. MSD	Skin (II)	NA	-	-	No	NA	NA	NA
26 (25)	Uncond. MFD	Skin (III)	Skin, lung	-	-	-	-	-	Chronic lung disease, oxygen dependent
27 (26)	Uncond. MSD	No	No	Negligible	Yes	No	0	73	Hearing loss
28 (25)	MAC MSD	NA	NA	-	-	-	-	-	-
29 (27)	Uncond. MSD	No	No	Negligible	No	No	0	60	Hearing loss, developmental delay
30 (24)	MAC MSD	No	No	Normal	Yes	Yes	61	< 50	None
31 (28)	Uncond. MFD	No	No	-	-	-	-	-	None

*NA* data not available

-: Data at > 24 months post-HSCT does not exist (because patient is deceased, or had a second procedure prior to 24 months after the first, or the patient does not yet have 24 months of follow-up at the end of the period defined for data collection)

^*^dATP results below 50umol/L are not reported with an exact value by the laboratory

*AI* autoimmune, *AIHA* autoimmune hemolytic anemia, *ADHD* attention deficit hyperactivity disorder

### Donor Chimerism Following HSCT

**Fig. 2:**
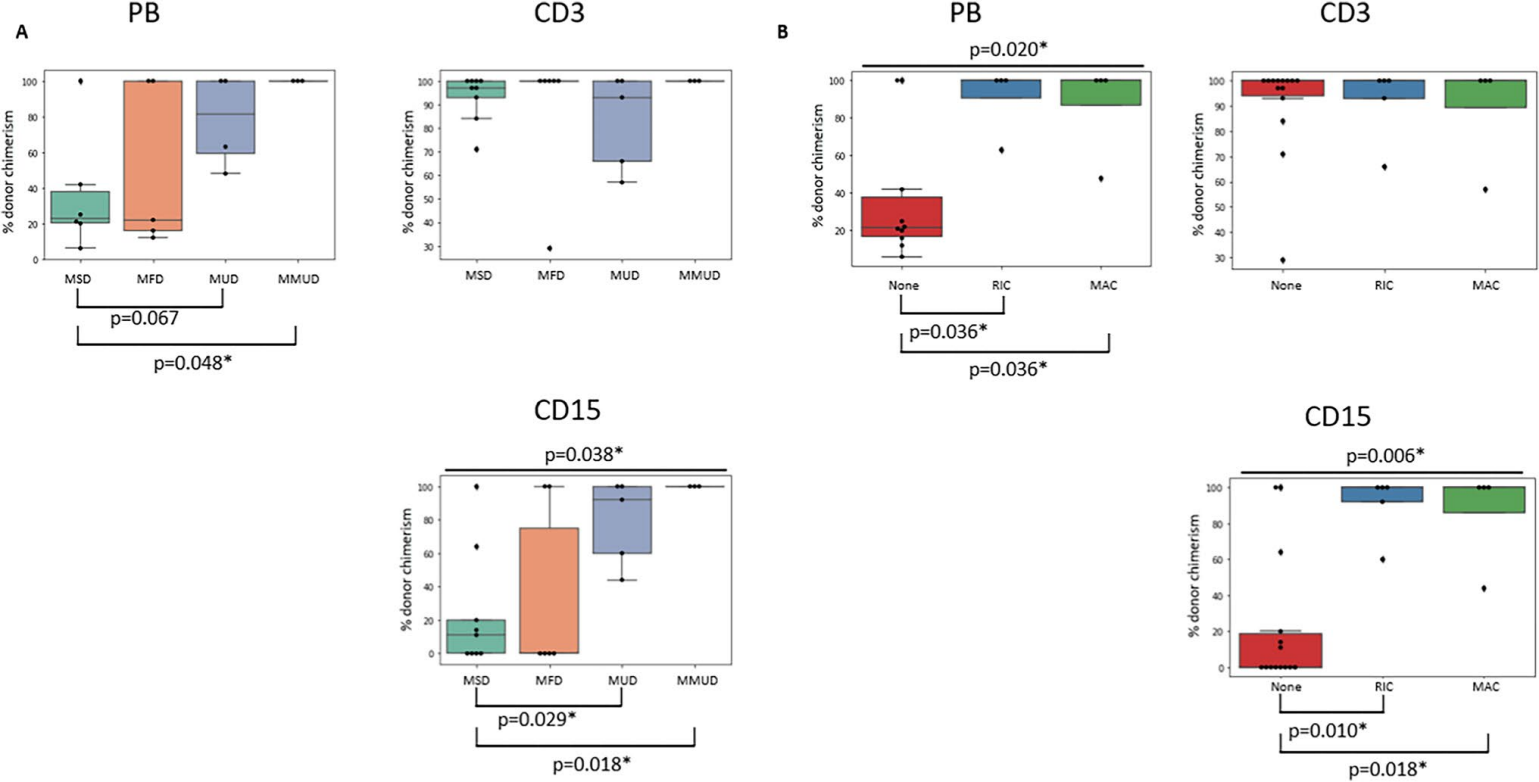
Donor chimerism after HSCT: Box plots showing the degree of donor chimerism at last follow-up in peripheral blood (PB), in CD3^+^ cells and CD15^+^ cells: **A** in relation to donor type for procedures using a MSD, MFD, MUD, or MMUD with n_MSD_ = 6, n_MFD_ = 5, n_MUD_ = 4, and n_MMUD_ = 3, respectively, in PB and n_MSD_ = 9, n_MFD_ = 6, n_MUD_ = 5, and n_MMUD_ = 3 in CD3^+^ cells and CD15^+^ cells; **B** in relation to intensity of conditioning for procedures without conditioning or using RIC or MAC with n_none_ = 10, n_RIC_ = 4, and n_MAC_ = 4, respectively, in PB and n_none_ = 14, n_RIC_ = 5, and n_MAC_ = 4 in CD3^+^ cells and CD15^+^ cells. Significant Kruskal–Wallis test results and (1-tailed) Mann–Whitney test results with *p* ≤ 0.05 have been indicated*, respectively, at the top and at the bottom of the box plots

Overall, across procedures, full donor chimerism in peripheral blood/whole blood (PB/WB) was achieved in 44.4% of patients at the last follow-up (at > 2 years post-HSCT) (Fig. 2A–B). Patients who underwent MUD and MMUD transplants achieved higher donor chimerism in PB/WB (77.8% and 100%, respectively) than patients who received MSD and MFD transplants (35.7 and 50%, respectively), with a statistically significant difference when comparing donor chimerism levels between MSD and MMUD procedures (*p* = 0.048) and a trend towards statistical significance when comparing MSD and MUD procedures (*p* = 0.067). These findings are in line with the intensity of conditioning. Indeed, after unconditioned cell infusions, average donor chimerism in PB/WB was 36.4% for 14 procedures with only 2 patients achieving full donor chimerism, whereas most patients achieved full donor chimerism after RIC and MAC with 90.75% and 87% average donor chimerism levels, respectively, at last follow-up (*p* = 0.036 when comparing chimerism levels in unconditioned procedures with those in RIC procedures, as well as MAC procedures). For the CD3^+^ compartment, a high rate of lymphoid engraftment was observed in MSD and MFD transplants (mean of 90.8% donor chimerism at > 2 years post-HSCT; ranging between 57 and 100%), despite the lack of conditioning. This is similar to the achieved CD3^+^ cell-specific engraftment after RIC and MAC (means of 89.2% and 91.8%, respectively). Donor chimerism levels in the CD19^+^ cell lineage were available for more than half of the patient cohort, with average 86% of donor engraftment after both unconditioned transplants and after RIC regimen, compared to 100% B cell engraftment after MAC (data not shown). In line with low donor chimerism in PB/WB, myeloid donor engraftment > 2 years after unconditioned HSCTs was significantly lower with an average level of 22.1%, compared to 90% and 86% after RIC and MAC, respectively (*p* = 0.010 and *p* = 0.018). Absent donor myeloid chimerism was seen in 8 patients (0% CD15^+^ donor chimerism in 7 patients and < 5% in 1 patient), all of whom underwent unconditioned procedures. Paired B cell and myeloid chimerism results were available for 8 patients, but differences of > 40% in the level of chimerism between B cell and myeloid lineages were only seen in 3 of these patients, all of whom received unconditioned procedures.

In summary, we find that levels of donor engraftment in CD19^+^ and CD15^+^, but not the CD3^+^ compartment, are lower in absence of conditioning.

### Immune Reconstitution

**Fig. 3:**
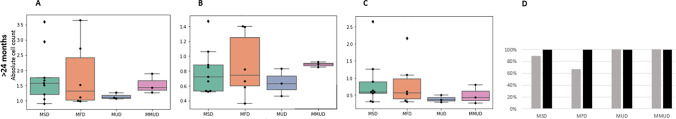
T cell immune recovery after HSCT: **A**–**C** Box plots showing levels of absolute T cell counts after HSCT in relation to donor type for procedures using a MSD, MFD, MUD, or MMUD with n_MSD_ = 9, n_MFD_ = 6, n_MUD_ = 3, and n_MMUD_ = 3 at last follow-up (> 24 months): Absolute counts (× 10^9^/L) of **A** CD3^+^ T cells, **B** CD4^+^ T cells, and **C** CD8^+^ T cells. Differences in cell counts between subgroups were not statistically different. **D** Proportion of HSCT procedures resulting in CD3^+^ recovery > 1000 cells/mm^3^ (gray) and CD4^+^ recovery > 300 cells/mm^3^ (black)

**Fig. 4:**
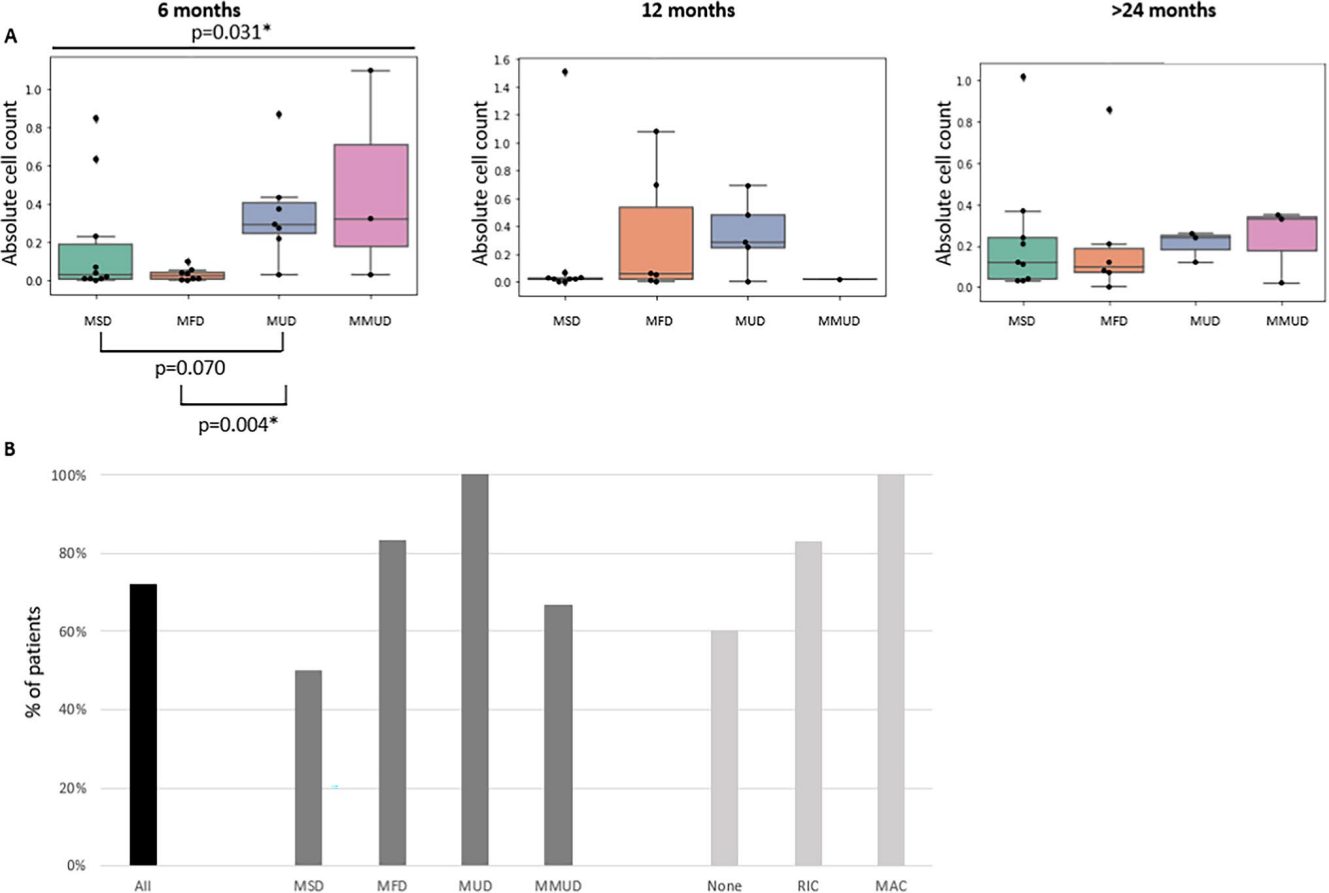
Humoral recovery after HSCT: **A** Levels of absolute B cell counts (× 10^9^/L) at 6 and 12 months and at last follow-up (> 24 months) after HSCT in relation to donor type for procedures using MSD, MFD, MUD, or MMUD with n_MSD_ = 10, n_MFD_ = 8, n_MUD_ = 7, and n_MMUD_ = 3, respectively, at 6 months, and n_MSD_ = 9, n_MFD_ = 6, n_MUD_ = 5, and n_MMUD_ = 1 at 12 months, and n_MSD_ = 9, n_MFD_ = 6, n_MUD_ = 3, and n_MMUD_ = 3 at last follow-up (> 24 months). Significant Kruskal–Wallis test results with *p* ≤ 0.05 have been indicated* at the top of the box plots. **B** Proportion of patients who have discontinued IgRT at 24 months post-HSCT in relation to donor type and conditioning regimen

Immune reconstitution was analyzed at 6 and 12 months after HSCT and at last follow-up beyond 24 months. The impact of transplant characteristics, in particular stem cell source and donor type, was investigated. Initially, at 6 months post-HSCT, patients receiving stem cells of bone marrow (BM) origin displayed higher absolute cell counts, specifically for CD3^+^ and CD8^+^ T cells, than patients who received cord blood (CB) and peripheral blood stem cells (PBSC) transplants. Beyond 6 months, there were no further significant differences in absolute T cell counts in relation to stem cell source (Supplementary Fig. 1). Over time, kinetics of T cell recovery were not affected by donor type, and similar absolute cell counts were observed over time for CD3^+^, CD4^+^, and CD8^+^ T cells in all donor settings (Fig. 3A–C), although CD8^+^ T-cell counts were significantly lower at 6 and 12 months following MUD and MMUD transplants (Supplementary Fig. 2A–C). At last follow-up, 89% and 67% of patients who underwent a MSD and MFD HSCT, respectively, had > 1000 CD3^+^ T cells/mm^3^. All patients who received a MUD or MMUD HSCT had > 1000 CD3^+^ T cells/mm^3^ at > 2 years post-HSCT. All patients, irrespective of donor type, had > 300 CD4^+^ T cells/mm^3^ at last follow-up (Fig. 3D). Data on naïve CD4^+^ and CD8^+^ T cell counts at last follow-up were limited but suggest that RIC and MAC, typically used in MUD and MMUD transplant procedures, did not affect naïve T cell numbers when compared with available counts from patients who did not receive conditioning (data not shown). Similarly, all patients having received MAC were found to have normal TREC levels at > 2 years post-HSCT (Table 3). In contrast, the assessment of TREC levels in 11 patients who had an unconditioned cell infusion showed that only one patient achieved a normal TREC level. CD19^+^ B cell recovery started earlier in patients who underwent MUD and MMUD transplant procedures (Fig. 4A). At last follow-up, CD19^+^ cell counts achieved similar levels in all different groups.

We also collected data on functional parameters of immune recovery post-HSCT (Table 3). For 23 patients assessed by T cell proliferative response to PHA by last follow-up, all but one patient displayed a normal PHA response. The T cell receptor (TCR) V beta repertoire was studied in 18 patients. Results were available for 11 patients after unconditioned cell infusion of whom 72.7% displayed a normal repertoire. 85.7% of the patients who received RIC/MAC showed a normal repertoire at last follow-up. The quality of humoral recovery was assessed by determining cessation of IgRT at 2 years post-HSCT (Fig. 4B). Overall, 72% of patients were able to stop IgRT (Table 3). For patients who received unconditioned MSD transplants, this fell below 50%. When assessing the possible impact of the intensity of the conditioning regimen, all patients who received MAC were able to discontinue IgRT compared to 83% of the patients with RIC regimens and 60% of those who underwent unconditioned procedures. All patients who stopped IgRT were able to produce specific responses to tetanus immunization; however, 26% of the patients demonstrated suboptimal responses to pneumococcal vaccine (with protective antibody titers > 0.35 μg/mL for less than 9 of the 13 pneumococcal serotypes tested) (data not shown). Upon immune reconstitution, patients were able to clear pre-existing infections and to cope with new infections, but long-term clinical follow-up emphasized the importance of non-infectious complications, which are summarized in Table 3.

### Use of Second Transplants

Primary graft failure was not seen in this cohort. Two patients (i.e., patients 7 and 11) received a second unconditioned stem cell infusion from the same MSD because of poor immune reconstitution more than a year after the first unconditioned infusion. The initial stem cell infusion for one of the patients was of CB origin. This patient developed 100% donor engraftment in the CD3^+^ compartment, but no myeloid engraftment, and metabolic detoxification was not successful. The second stem cell infusions in both patients were of BM origin, and both patients achieved good immune reconstitution. Due to poor donor engraftment and immune reconstitution, 3 patients (i.e., patients 15, 24, and 25) required second conditioned HSCT procedures. One patient achieved full donor T cell chimerism after the initial RIC MUD procedure but failed to gain myeloid engraftment and displayed poor immune reconstitution. He received a second MUD transplant after RIC and achieved full donor engraftment and good immune reconstitution. The second patient did not achieve stable donor engraftment after a first unconditioned MSD infusion. A second HSCT after MAC from another MSD was successful with full donor engraftment and good immune reconstitution. Due to his poor clinical condition, the 3^rd^ patient first received an unconditioned MFD cell infusion. He developed GVHD, and persistent immunosuppressive treatment affected his immune reconstitution. He subsequently underwent a TCRαβ-depleted 2^nd^ HSCT from a MSD after MAC in an attempt to treat refractory GVHD but died 55 days later.

### Metabolic Detoxification and ADA Activity

Finally, we investigated the metabolic correction after HSCT by assessing dATP levels and ADA activity at last follow-up at 2 or more years post-HSCT. Results were available for 17 patients (Table 3). Only 3 patients had elevated dATP levels at last follow-up (60–157 μmol/L), all of whom had undergone unconditioned infusions and remain off ERT. Normal ADA activity was reported as > 40 nmol/mg Hb/h. In our cohort, results ranged between 0 and 130 nmol/mg Hb/h with an average result of 32.9 nmol/mg Hb/h. The median value was 27 nmol/mg Hb/h with only 6 patients (35.3%) achieving normal ADA activity. Interestingly, no patient achieved normal ADA activity after an unconditioned cell infusion, whereas all patients after RIC/MAC transplant procedures did. Low ADA activity was specifically associated with poor myeloid donor engraftment (Supplementary Fig. 3).

## Discussion

Thanks to optimized supportive care over the past years, outcome following allogeneic HSCT has improved, as patients enter transplant in a better state of health. Further improvements are expected, thanks to the wider implementation of universal newborn screening (NBS) for SCID, allowing for earlier diagnosis and initiation of protective measures, including prophylactic antimicrobials, IgRT, and isolation [33]. Recently updated guidelines for the management of ADA-SCID recommend that all patients should also receive ERT upon diagnosis [20]. This should be followed as soon as feasible by definitive treatment with either of 2 available options: allogeneic HSCT or ex vivo-corrected autologous HSC-GT. In particular, the most recent consensus statement recommends that HSC-GT and MSD/MFD HSCT (without RIC) are seen as equal therapeutic options [20]. Previous studies have reported only smaller numbers of patients who underwent unconditioned infusions [27–30, 32], but this recommendation is supported by the findings from the largest multicenter study to date, which in addition to better OS reports faster T cell immune recovery after MSD/MFD transplant procedures, as well as good humoral recovery even though most were unconditioned procedures [21]. Overall, detailed analysis of the long-term outcome after HSCT in ADA deficiency is still limited. Moreover, there are no reports focused on cohorts of ADA-deficient patients who have been transplanted more recently, which would constitute a more appropriate comparison for current GT studies. GOSH is one of the biggest pediatric bone marrow transplantation centers worldwide with one of the largest cohorts of transplanted ADA-deficient SCID patients. We thus undertook a single-center study reviewing the immunological outcome of all HSCT procedures performed in ADA-deficient patients since 2000. To our knowledge, this is the largest single-center cohort, with 28 ADA-deficient patients, who collectively underwent 31 HSCT procedures.

More than half of these procedures were unconditioned MSD/MFD transplants. GVHD complicated 45% of all procedures and, similarly, 47% of the procedures using MSDs and MFDs. In some patients, GVHD was less than grade II, but any degree of GVHD is undesirable in this population of patients. As in previous studies, we observed a trend of improved OS after unconditioned procedures (Fig. 1). However, four of these patients (22%) required a second intervention to achieve long-term engraftment and immune reconstitution. Specifically, two patients received a second unconditioned infusion of whole bone marrow from the same donor, and two patients underwent second myeloablative HSCT procedures from MSDs, after first unconditioned procedures with a different MSD and a MFD, respectively. The reasons for this higher failure rate after unconditioned MSD/MFD remain poorly understood. These patients received ERT prior to HSCT, and one hypothesis is that the level of immune function established by ERT at the time of HSCT may contribute to rejection or non-engraftment of donor cells. Standard practice in our center is to initiate ERT at diagnosis and, in general, stop ERT 1 month prior to proceeding to HSCT. However, detailed data on when exactly ERT was discontinued is not available, and therefore, we could not address whether the timing of ERT discontinuation impacts hematopoietic engraftment after HSCT. Overall, our data shows that, alternatively, the use of RIC, even in MSD/MFD procedures, could be used to deplete the cells resulting from the immune reconstitution due to ERT.

Independently of the type of transplant procedure, whether unconditioned MSD/MFD procedures or not, we observe similar absolute T and B cell counts at > 2 years post-HSCT (Figs. 3 and 4 and data not shown). However, long-term follow-up of our patient cohort highlights that, whereas high levels of lymphoid donor engraftment are achieved in the CD3^+^ compartment even in the absence of conditioning, donor engraftment in CD19^+^ and CD15^+^ compartments is significantly better in conditioned procedures than in unconditioned ones (Fig. 2 and data not shown). Previous observations suggested that there may be a selective advantage for donor B cell engraftment [21], but in more than half the patients in our cohort, we observed a good correlation between B cell and myeloid engraftment when paired results were available. Interestingly, approximately 30% of the patients in our cohort had not yet discontinued IgRT at 2 years post-HSCT. All of these patients, except for one, had undergone an unconditioned procedure and were found to have absent myeloid engraftment. These patients also show poor metabolic correction with lower ADA activity at their last follow-up appointment than the other patients in the cohort. All patients with absent ADA activity and with elevated dATP levels after HSCT (all unconditioned with low myeloid chimerism) remained on IgRT at 2 years post-HSCT, suggesting that insufficient myeloid engraftment is associated with inadequate metabolic correction post-HSCT. The lack of conditioning thus comes at a significant cost.

Previous studies have already reported that lymphoid and humoral immune reconstitution in SCID patients post-HSCT are improved using conditioning procedures [34, 35]. Additionally, a recent study showed that the use of a RIC regimen for non-malignant HSCT procedures using HLA-identical donors, including MSDs and MFDs, can achieve sustained myeloid engraftment with a low incidence of GVHD and conditioning-related toxicities [36]. A case report of 2 ADA-deficient siblings treated by MSD HSCT, one unconditioned and one after RIC, already proposed the need to explore the use of RIC in HSCT procedures from HLA-matched donors to improve long-term immune reconstitution with better myeloid engraftment and metabolic recovery in ADA SCID specifically as well [37]. In our cohort, all first HSCT procedures using a MSD/MFD were unconditioned. Nevertheless, we postulate that routine use of RIC in ADA SCID patients receiving a MSD/MFD transplant may improve the long-term outcome of these patients by achieving better engraftment.

ADA-deficient SCID is rare, and the cohort size of our study remains small. To further confirm the potential role for RIC in the long-term outcome for ADA SCID patients after MSD and MFD transplants, this type of procedures must continue to be performed in the context of multicenter clinical trials. This is particularly important for ADA-deficiency given that, unlike other forms of SCID, its management includes multiple treatment options. Specifically, more than 100 ADA-deficient patients have been treated with HSC-GT and the overall survival is 100% [23–26, 38] (unpublished data and personal communications), including from the use of an approved treatment in Europe using a gammaretroviral vector licensed as Strimvelis® which is available in a single center [38, 39]. This treatment requires RIC with single agent low-dose busulfan (dose range ~ 4–5 mg/kg for a target AUC of 20 mg/L × hour) to achieve engraftment of gene-corrected cells, immune recovery, and metabolic detoxification [23–25, 40, 41]. This conditioning regimen is typically well tolerated and is milder than RIC regimens used in allo-HSCT. Until recently, no serious adverse events, nor genotoxic insertional mutagenesis, had been reported in the largest cohort of ADA-deficient patients treated with HSC-GT [42]. A patient with Strimvelis® has very recently been diagnosed with T cell leukemia [43]. Causality is under investigation. Global clinical trials using a lentiviral approach have demonstrated extremely promising results ([44], unpublished data and personal communication) and may offer a further treatment option in the near future. No insertional oncogenesis has been reported after lentiviral vector-based GT, in any indication [45].

In conclusion, long-term outcome data is evolving, and further monitoring is required. Longitudinal data collection in appropriately powered prospective trials will inform treatment optimization for future patients and will be the basis for updates to treatment guidelines. This will become increasingly important as newborn screening programs are introduced more widely, given several therapeutic approaches exist to treat ADA SCID. We propose that, if accessible, HSC-GT should possibly be considered a preferred first-line treatment to HSCT, even when a MSD or MFD is available, as the autologous nature of this procedure abrogates any risk of alloreactivity, in particular if further studies support the recommendation for routine use of RIC for all HSCT procedures using HLA-identical donors in order to achieve better myeloid engraftment, humoral immune recovery, and metabolic correction.

## Supplementary Information

Below is the link to the electronic supplementary material.

Supplementary file1 (DOCX 231 KB)

## Data Availability

Requests for data can be addressed to c.booth@ucl.ac.uk.
