# Supplementary material for: Long-Term Immune Recovery After Hematopoietic Stem Cell Transplantation for ADA Deficiency: a Single-Center Experience
Source: J Clin Immunol. 2021 Oct 16;42(1):94–107. doi: 10.1007/s10875-021-01145-w (PMC8821083; doi:10.1007/s10875-021-01145-w)

**Supplementary Table 1: Mortality details:**

| **Cause of death** | **Time post HSCT** |
| --- | --- |
| Patient 1: Pre-existing disseminated CMV disease | 16 days |
| Patient 3: Sepsis | 56 days |
| Patient 20: Lung cGvHD | 3.6 years |
| Patient 25: Respiratory failure (previous lung GvHD) | 55 days |

**Supplementary Figure 1:** Box plots showing levels of absolute T cell counts at 6 and 12 months and at last follow-up (>24 months) after HSCT in relation to stem cell source: Absolute counts (x10^9^/L) of A. CD3^+^ T cells, B. CD4^+^ T cells and C. CD8^+^ T cells.

**
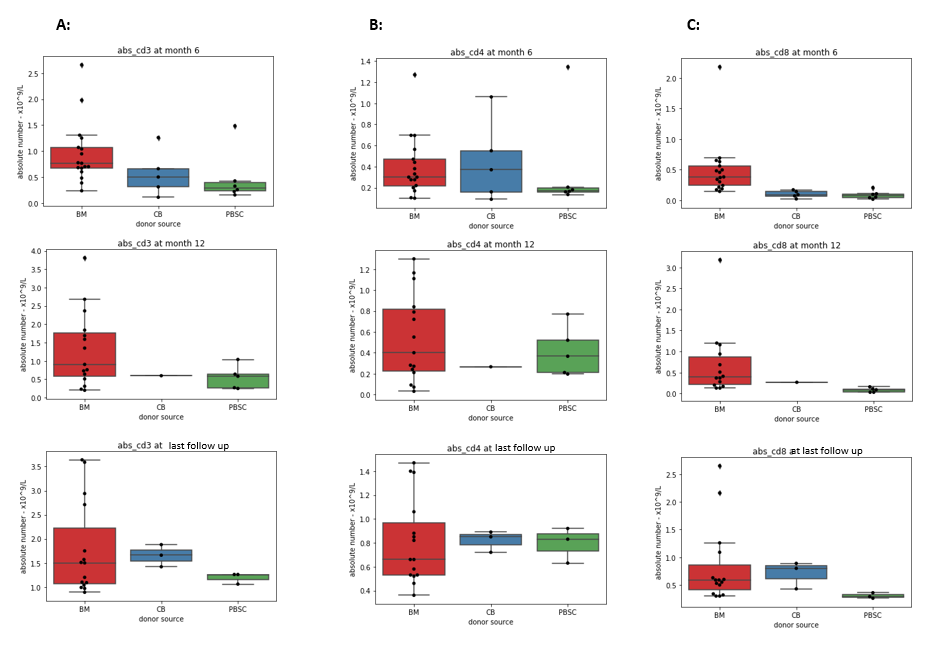
**

**Supplementary Figure 2: T cell immune recovery after HSCT: 3A-C:** Box plots showing levels of absolute T cell counts after HSCT in relation to donor type for procedures using a MSD, MFD, MUD or MMUD with n_MSD_=10, n_MFD_=8, n_MUD_=7 and n_MMUD_=3 respectively at 6 months, and n_MSD_=8, n_MFD_=7, n_MUD_=5 and n_MMUD_=1 at 12 months: Absolute counts (x10^9^/L) of **A.** CD3^+^ T cells, **B.** CD4^+^ T cells and **C.** CD8^+^ T cells. **3D:** Proportion of HSCT procedures resulting in CD3^+^ recovery >1000 cells/mm^3^ (grey) and CD4^+^ recovery >300 cells/mm^3^ (black).

**
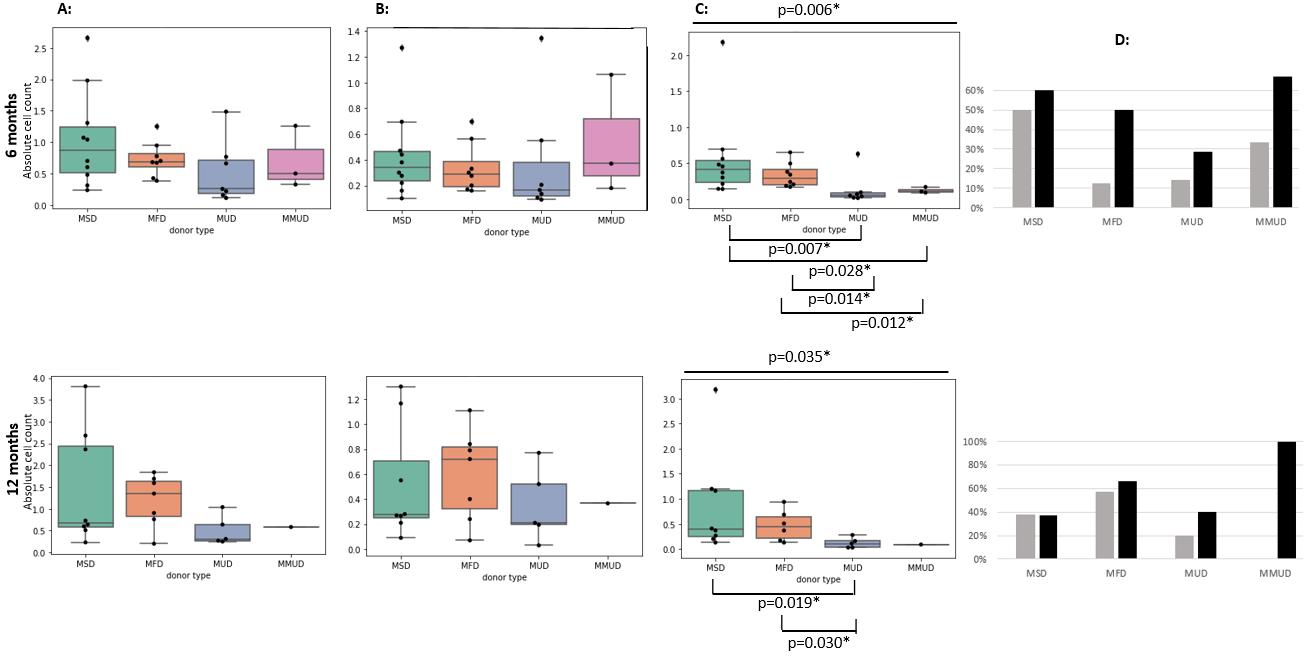
**

**Supplementary Figure 3: Poor metabolic recovery after HSCT is associated with poor myeloid donor engraftment:** Average % donor chimerism in patients with ADA activity below or above 40nmol/mg Hb/h.

% donor chimerism


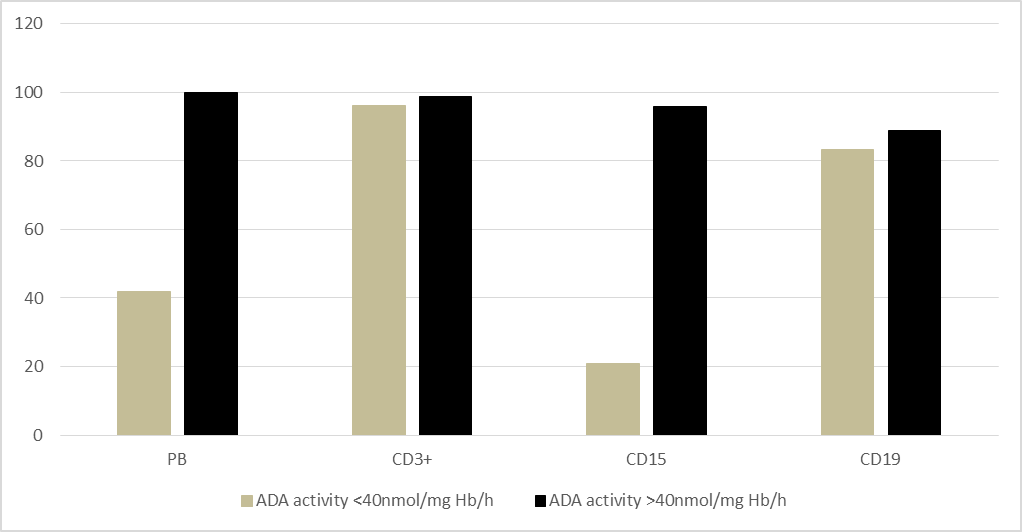

Supplement: Supplementary file 1 — Supplementary file1 (DOCX 231 KB) [file 10875_2021_1145_MOESM1_ESM.docx]
